# Editorial to Special Issue—Food Brewing Technology and Brewing Microorganisms

**DOI:** 10.3390/foods12173324

**Published:** 2023-09-04

**Authors:** Yanru Chen, Guiming Fu, Jinjing Wang, Wenqin Cai

**Affiliations:** 1 State Key Laboratory of Food Science and Resources & College of Food Science and Technology, Nanchang University, Nanchang 330047, China; 2 International Institute of Food Innovation, Nanchang University, Nanchang 330299, China; 3 Key Laboratory of Industrial Biotechnology, Ministry of Education, School of Biotechnology, Jiangnan University, Wuxi 214122, China

Food brewing technology is an important technology in the modern worldwide food industry, which uses the specific traits of microorganisms to produce food by traditional or modern engineering techniques. The products produced by food brewing technology have spread all over the world. Among them, alcoholic products (Chinese *Baijiu*, fruit wine, rice wine, beer, whisky, etc.), as important fermented food, have been loved by consumers all over the world. This Special Issue includes a total of eleven articles, and nine of them involve research on brewing technology of alcoholic products and brewing microorganisms. This phenomenon indicates that the alcoholic products industry occupies an important position in the field of food fermentation. Therefore, in response to the research content of these nine articles, this editorial mainly explores the research hotspots in the field of alcohol brewing.

Organic acid is one of the main flavor substances in fruit wine. An appropriate amount of organic acids can give fruit wine a smooth taste and have certain antibacterial effects, but the high organic acid content gives people an unpleasant experience, which seriously affects the sensory quality of fruit wine. At present, the high content of organic acids in fruit wine has become one of the important obstacles restricting the further development of the fruit wine brewing industry. The usual method to alleviate excessive acidity is deacidification by chemical or physical processing. Unfortunately, the methods of chemical or physical are not effective in all cases and may reduce the stability of the wine. However, a biological deacidification method, such as malolactic fermentation (MLF), can significantly reduce the concentration of malic acid and total acidity in wines, without negatively impacting the flavor quality. Yang et al. (2022) found that an *Oenococcus oeni* GF-2 strain had good MLF performance and high glucosidase activity. MLF significantly decreased the concentration of organic acids by 29.7% and promoted the accumulation of aromatic esters, higher alcohols, and terpenoids. MLF wine achieved the highest sensory scores for aroma, taste, and overall acceptability [1]. This study indicated that MLF with *O. oeni* GF-2 had great potential to markedly improve the aroma and taste of commercial pear–kiwifruit wine. Meantime, the biological deacidification method may gradually replace chemical or physical deacidification methods in the future, becoming the main method to reduce the organic acid content in fruit wine.

*Baijiu* and rice wine are traditional Chinese alcoholic products with a long history of brewing. During the brewing process, a variety of brewing microorganisms use sugar, cellulose and other raw materials to grow and produce more than 100 flavor substances, which can form different flavor types of *Baijiu* and rice wine. Among them, the brewing microorganisms mainly come from *Jiuqu*, pit mud and fermented grains, and together affect the brewing process and flavor quality of *Baijiu* and rice wine. Therefore, it is of great significance for standardizing the brewing industry of *Baijiu* and rice wine in the future to explain the types of brewing microorganisms and their metabolic mechanisms in the brewing process of *Baijiu* and rice wine. At present, Pu and Yan (2022) found differences in fungal community structures and physicochemical properties in pit mud samples from different spatial positions within *Nongxiang Baijiu* fermentation cellars, revealing unique characteristic multidimensional pit mud fungal community profiles [2]. Chen et al. (2023) also reported on the correlation between fungal biomarkers in *Jiuqu* and the style of corresponding rice wine from the rim of the Sichuan Basin in Southwest China [3].

In addition, the research emphasis on *Jiuqu* microorganisms also included the metabolic functions of specific microbial communities. For example, the fermentation functional microbiota (FFM) and alcohol functional microbiota (AFM) were obtained by correlating the fermentation ability and alcohol production ability from *Jiuqu* microbiota of *Nongxiang Baijiu*, and function predictions indicated that fermentation functional bacterial microbiota was active in metabolic functions related to product synthesis and transport, and the alcohol functional bacterial microbiota was very active in raw material utilization and its own metabolic synthesis [4]. Meantime, understanding the macrogenome of the microbiota is also necessary to classify and obtain a given community’s genomic and functional details. The carbohydrate metabolism is the most critical functional module in *Jiuqu* fermentation. Almost all flavor compounds in *Jiuqu*, including alcohols, esters, phenols, and acids, are derived from the carbohydrate metabolism during fermentation, which is closely related to the growth and material exchange of microorganisms in the fermentation environment. Zhang et al. (2022) studied the changes in microbial functions and the relationship between carbohydrate metabolism-related functional genes and extracellular enzyme activity during the *Jiuqu* fermentation of *Nongxiang Baijiu*. The results showed that the pathways related to metabolites were less in the early fermentation stage, but more in the middle and late stages. The expression levels of functional genes related to pyruvate metabolism, glyoxylate and dicarboxylate metabolism, and propanoate metabolism were relatively high in the early and late stages of fermentation, while the expression levels of these genes in start and cross stages were relatively low. The study also found that amino sugar and nucleoside sugar metabolisms were dominant in the middle stage of fermentation [5]. The above four articles revealed the microbial communities and related mechanism pattern of *Jiuqu* and pit mud in *Baijiu* and rice wine, which provided the theoretical basis for designing effective strategies to manipulate microbial consortia to improve the quality of Chinese *Baijiu* and rice wine.

Of note, the natural evolution of pit mud (PM) microbiota is very slow and needs more than 20 years of uninterrupted domestication to produce high-quality *Nongxiang Baijiu*. Therefore, it is an important reason for the development of artificial pit mud (APM) manufacturing technology. APM manufacturing is a directional evolution process of functional consortia under anaerobic conditions. Traditionally, the APM improvement studies mainly focused on the different formulas, functional strains, and cultivation patterns and times due to the scarcity of high-quality PM resources and the frequent degradation of aged PM. The effects of *Jiuqu* on APM quality received little attention. Here, Mu et al. (2022) used two types of APM manufactured by adding fortified *Jiuqu* (FJ) and conventional *Jiuqu* (CJ). The results showed that FJ altered the prokaryotic communities rather than the fungal ones. Correlation analysis suggested that these variations in community structure promoted the formation of hexanoic acid, butyric acid, and the corresponding ethyl esters, and inhibited the formation of lactic acid and ethyl lactate, thus improving the flavor quality of the APM [6]. Specifically, this study highlights the importance of *Jiuqu* in APM cultivation and may contribute to the optimization of the APM manufacturing technology and the production of high-quality *Nongxiang Baijiu*.

In addition to analyzing the overall microbial community structure and functional effects in *Baijiu* and rice wine, isolation, identification, and purification of microorganisms during the brewing process, as well as the exploration of the related functions, applications, and tolerance mechanisms of single microorganisms, are also the main subjects of current research on brewing microorganisms. For example, *Clostridium* is the key bacteria that inhabit the pit mud in a fermentation cell during the fermentation of *Nongxiang Baijiu*. Their activities in the process of *Baijiu* fermentation are closely related to the niches of pit mud and cells. After multiple rounds of underground fermentation, *Clostridium* has been domesticated and adapted to the environment. However, the mechanisms of clostridia succession in the pit mud and how they metabolize nutrients in grains are not clear. Herein, 15 *Clostridium* species including three firstly reported ones (*Clostridium tertium*, *Clostridium pabulibutyricum* and *Clostridium intestinale*) were isolated from the pit mud of *Nongxiang Baijiu*. Eighty-one percent of these *Clostridium* strains were motile, and most of them showed chemotaxis to organic acids, glutathione, saccharides and lactic acid bacteria. In a simulated *Baijiu* fermentation system, *Clostridium* migrated from pit mud to fermented grains with the addition of chemokine lactic acid, resulting in the production of acetic acid and butyric acid [7]. This study helps us to understand the succession mechanism of *Clostridium* in pit mud, and provides a reference for the regulation of lactic acid level in fermented grains during *Baijiu* fermentation.

A large number of aroma-producing yeasts are found in the fermentation process of *Baijiu*, and the application of these yeasts in fruit wine fermentation can help improve the flavor of fruit wine. Also, mixed fermentation of fruit wine by *Saccharomyces cerevisiae* and aroma-producing yeasts is an important means to change the single flavor substances of fruit wine. Cai et al. (2022) screened three aroma-producing yeasts with high ester yields from the fermentation process of special-flavor *Baijiu*, and applied them to the simulated fermentation process of blueberry wine. The results showed that the content of flavor substances in the blueberry wine fermented with *S. cerevisiae* and *Candida glabrata* was the highest, indicating that *C. glabrata* had the potential to create blueberry wine with a unique flavor [8].

The brewing process of alcoholic products has always been a complex system with dynamic environmental changes of physicochemical factors. With the increase in ethanol concentration during the brewing process, yeast is susceptible to ethanol stress, which affects the fermentation efficiency, leading to the decline of quality and flavor profiles in the finished alcoholic products. Chen et al. (2022) found that ethanol stress could inhibit the growth and ester production metabolism of *Pichia anomala*. The ethanol stress might cause the reactive oxygen species burst in *P. anomala*, thereby promoting the accumulation of malondialdehyde content in the cell membrane, destroying the integrity of the cell membrane and leading to the leakage of intracellular nutrients and electrolytes. Finally, the growth and ester production ability of *P. anomala* was inhibited [9].

All in all, these nine articles each have their own highlights, revealing the application of the biological deacidification method in fruit wine, the structure and function of microbial communities during *Baijiu* and rice wine fermentation, and the diversity of research directions in brewing microorganisms. This has important theoretical and practical significance for the development of the alcoholic products brewing industry in the future.

In addition, the abstracts of the other two articles in this Special Issue are as follows [10,11]:

(1) In this study, the author obtained a mutant strain of *Saccharomyces pastorianus* G03H8 with the potential to greatly reduce the cost of RNA production and shorten the fermentation cycle via ARTP mutagenesis technology. The highest RNA content of mutant strain G03H8 increased by 40% compared with the parental strain G03. The RNA production and DCW of G03H8 reached 3.58 g/L and 60.58 g/L in fed-batch with molasses flowing. In addition, the reason for high RNA production in mutant strain G03H8 is closely related to the ribosome bio-genesis, yeast meiosis, RNA transport, and longevity regulating pathways.
(2) In order to complement the mechanism of asexual sporulation of *Antrodia cinnamomea* in submerged fermentation and provide a theoretical basis to further improve the sporulation, in this study comparative transcriptomics was used to reveal the regulatory mechanism underlying asexual sporulation of *A. cinnamomea* induced by nutrient limitation in submerged fermentation. It was found that the signals of nutrient limitation (including carbon and nitrogen starvation) were firstly responded to by the corresponding sensors, such as Rco-3. Then, the sensors cause an effect on the downstream genes, such as *areA*, *tmpA*, *gluC*, and *chsD*. These genes play a direct or indirect role in enhancing the expression of the genes in the central signaling pathway, such as *flbD*, *brlA*, and *wetA*, thereby promoting the sporulation.

We hope that this Special Issue will be useful for all readers in terms of the novel information it provides on food brewing technology and brewing microorganisms, helping us better promote the healthy development of the fermentation industry.
